# Deep learning radiomics based on multimodal imaging for distinguishing benign and malignant breast tumours

**DOI:** 10.3389/fmed.2024.1402967

**Published:** 2024-07-05

**Authors:** Guoxiu Lu, Ronghui Tian, Wei Yang, Ruibo Liu, Dongmei Liu, Zijie Xiang, Guoxu Zhang

**Affiliations:** ^1^College of Medicine and Biological Information Engineering, Northeastern University, Shenyang, Liaoning, China; ^2^Department of Nuclear Medicine, General Hospital of Northern Theater Command, Shenyang, Liaoning, China; ^3^Department of Radiology, Cancer Hospital of China Medical University, Liaoning Cancer Hospital and Institute, Shenyang, Liaoning, China; ^4^Department of Ultrasound, Beijing Shijitan Hospital, Capital Medical University, Beijing, China; ^5^Biomedical Engineering, Shenyang University of Technology, Shenyang, Liaoning, China

**Keywords:** deep learning, radiomics, multimodality imaging, breast tumours, deep learning radiomics, MRI, Mammography, Ultrosonography

## Abstract

**Objectives:**

This study aimed to develop a deep learning radiomic model using multimodal imaging to differentiate benign and malignant breast tumours.

**Methods:**

Multimodality imaging data, including ultrasonography (US), mammography (MG), and magnetic resonance imaging (MRI), from 322 patients (112 with benign breast tumours and 210 with malignant breast tumours) with histopathologically confirmed breast tumours were retrospectively collected between December 2018 and May 2023. Based on multimodal imaging, the experiment was divided into three parts: traditional radiomics, deep learning radiomics, and feature fusion. We tested the performance of seven classifiers, namely, SVM, KNN, random forest, extra trees, XGBoost, LightGBM, and LR, on different feature models. Through feature fusion using ensemble and stacking strategies, we obtained the optimal classification model for benign and malignant breast tumours.

**Results:**

In terms of traditional radiomics, the ensemble fusion strategy achieved the highest accuracy, AUC, and specificity, with values of 0.892, 0.942 [0.886–0.996], and 0.956 [0.873–1.000], respectively. The early fusion strategy with US, MG, and MRI achieved the highest sensitivity of 0.952 [0.887–1.000]. In terms of deep learning radiomics, the stacking fusion strategy achieved the highest accuracy, AUC, and sensitivity, with values of 0.937, 0.947 [0.887–1.000], and 1.000 [0.999–1.000], respectively. The early fusion strategies of US+MRI and US+MG achieved the highest specificity of 0.954 [0.867–1.000]. In terms of feature fusion, the ensemble and stacking approaches of the late fusion strategy achieved the highest accuracy of 0.968. In addition, stacking achieved the highest AUC and specificity, which were 0.997 [0.990–1.000] and 1.000 [0.999–1.000], respectively. The traditional radiomic and depth features of US+MG + MR achieved the highest sensitivity of 1.000 [0.999–1.000] under the early fusion strategy.

**Conclusion:**

This study demonstrated the potential of integrating deep learning and radiomic features with multimodal images. As a single modality, MRI based on radiomic features achieved greater accuracy than US or MG. The US and MG models achieved higher accuracy with transfer learning than the single-mode or radiomic models. The traditional radiomic and depth features of US+MG + MR achieved the highest sensitivity under the early fusion strategy, showed higher diagnostic performance, and provided more valuable information for differentiation between benign and malignant breast tumours.

## 1 Introduction

Breast cancer is the most prevalent cancer and the second leading cause of cancer-related deaths among women in the United States (1). In 2023, an estimated 55,720 women were diagnosed with carcinoma *in situ*, whilst 297,790 were diagnosed with invasive carcinoma, and 43,170 women died from breast cancer (2). Early diagnosis and classification are critical for effective treatment. Currently, many imaging modalities, such as ultrasonography (US), mammography (MG), and magnetic resonance imaging (MRI), are commonly used for the classification and diagnosis of breast cancer (3). MG is the predominant tool used for breast cancer screening (4–6), showing high sensitivity for calcification, but its low specificity is one of its limitations. Consequently, a large number of unnecessary biopsies are carried out, leading to healthcare resource waste and stress for patients (7, 8). These disadvantages have led to increased use of other adjunct imaging modalities in clinical practise, including US and MRI (9). US can effectively distinguish between cysts and solid masses and is more sensitive in dense breasts than MG (10). As an adjunct to MG, US provides highly accurate breast mass information and facilitates annotations (11, 12), but it often misses certain types of breast masses, such as invasive micropapillary carcinoma, ductal carcinoma *in situ*, invasive lobular carcinoma, fat-surrounded isoechoic lesions, heterogeneous echoic lesions with heterogeneous backgrounds, subareolar lesions, and deep lesions in large breasts. Additionally, lesions may be missed due to poor operator skills (12–15). MRI, which has high sensitivity, supports multiplanar scanning and 3D reconstruction, allowing for better visualisation of breast lesion size, shape, and location (16). MRI is valuable for screening high-risk individuals, diagnosing occult cases, staging, and assessing the response to chemotherapy (17, 18). However, MRI scans are expensive, and the examination requires more time than other tests (19).

Early and precise detection of malignant breast lesions is crucial for timely intervention and improvement of patient prognosis. Conventional diagnostic methods such as US, MG, and MRI are available but have inherent limitations, including indistinct boundaries, false-positive results, and potential sampling errors. In recent years, deep learning radiomics (DLR) in breast cancer has gained attention as a promising field (20, 21). Although deep learning (DL) models have achieved considerable progress in the automatic segmentation and classification of breast cancer (22, 23), data on how they are improving the overall management of breast cancer, starting from screening to diagnosis and ultimately to survival, are lacking (24). MG, US, and MRI are routinely used during breast cancer screening and are commonly used to identify and characterise breast lesions and guide biopsy. Several studies have focussed on MG and US. Cruz et al. (25) proposed a method consisting of different steps, including segmentation and extraction of deep learning features performed by a CNN—specifically, DenseNet 201. They analysed deep learning and handcrafted features during the fusion stage and then applied several classifiers (XGBoost, AdaBoost, and multilayer perceptron) based on stochastic measures. Ultimately, they achieved strong performance in multimodal imaging studies (US and MG). Lamb et al. (26) reported a higher cancer detection rate for patients who underwent breast screening by MRI than for patients identified as high risk with the traditional risk model using a retrospective mammogram-based model of 2,168 women. Natalia et al. (27) tested three different clinical imaging modalities (dynamic contrast-enhanced MRI, full-field digital mammography, and ultrasound) by pretraining a CNN and fusing it with deep learning methods for radiomic computer-aided diagnosis. They found that compared to previous breast cancer methods, computer-aided diagnosis methods achieved better performance in distinguishing between malignant and benign lesions. However, open questions remain on how to use the DL risk assessment model in clinical practise, and few studies have focussed on multimodality imaging based on deep learning and radiomics with MG, US, and MRI.

The aim of this study was to develop a comprehensive deep learning radiomic framework utilising multimodal imaging data, including MG, US, and MRI data. By integrating deep learning radiomic technology with multimodal imaging, complementary information from different imaging modes can be leveraged to fully characterise the imaging features of breast tumours, thereby achieving a greater differential diagnosis capability for benign and malignant tumours than single-mode radiomics, which will ultimately lead to a reduction in unnecessary biopsies.

## 2 Materials and methods

### 2.1 Patient population

**Figure 1:**
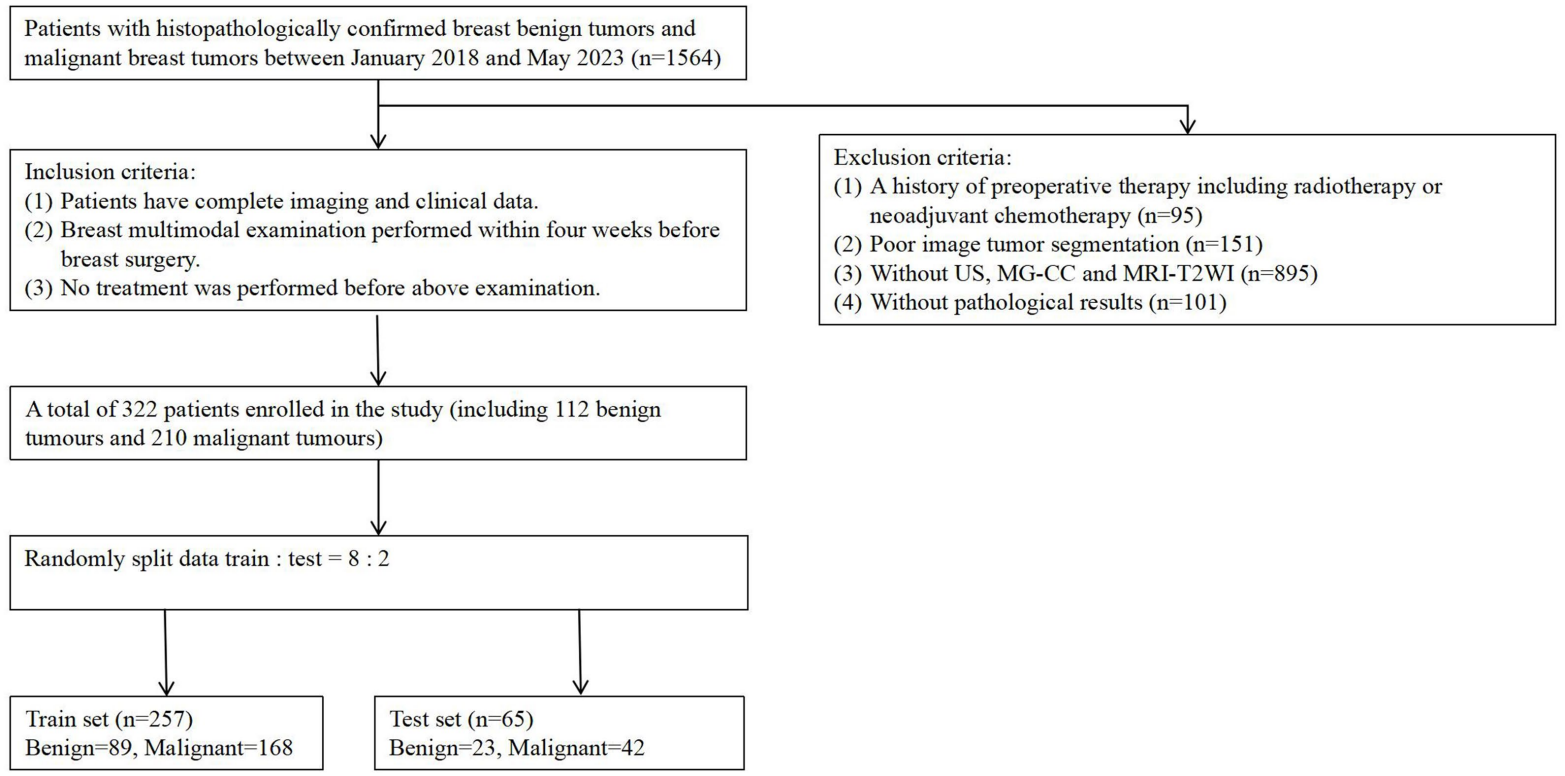
Flowchart of patient recruitment. A total of 322 patients enrolled in the study (including 112 benign tumors and 210 malignancy tumors), with the training set (*n* = 257) consisting of 89 benign tumors and 168 malignant tumors.

This retrospective study obtained approval from the institutional review board of our hospital (Approval No. Y(2404)-030), and the requirement for informed consent was waived. This study enrolled 1,564 female patients who preoperatively underwent multimodality (US, MG, and MRI) examinations at our centre between January 2018 and May 2023. The inclusion criteria were (a) complete imaging and clinical data availability, (b) multimodality breast examination performed within 4 weeks before breast surgery, and (c) no treatment performed before the aforementioned examination. The exclusion criteria were as follows: (a) a history of preoperative therapy, including radiotherapy or neoadjuvant chemotherapy; (b) poor image tumour segmentation due to blurred boundaries; (c) missing US, MG, and MRI data; and (d) no available pathological results. Ultimately, 322 patients (112 with benign breast tumours and 210 with malignant breast tumours). The training cohort included 257 patients (89 with benign and 168 with malignant breast tumors) enrolled in the training cohort and 65 patients (23 with benign breast tumours and 42 with malignant breast tumours) enrolled in the internal testing cohort. The enrolment process is shown in Figure 1.

### 2.2 Image acquisition

Each patient’s multimodality imaging examinations were as follows: MG-CC, MRI-T2WI, and US. In all patients, routine digital mammography was performed with the Hologic Selenia Dimensions system using standard, craniocaudal (CC), and mediolateral (MLO) views, and we analysed the former images. Routine ultrasound, including Doppler US, was performed using Philips IU22 and EPIQ7 instruments with 12–5-MHz transducers. All contrast-enhanced MRI examinations were performed on a 3.0 T MR system (Skyra, Siemens Healthcare, 3.0 T GE Discovery MR750) in the prone position with no breast compression using a dedicated four-channel breast coil and the following sequences: T2-weighted imaging (T2WI), dynamic contrast-enhanced (DCE) imaging, and diffusion-weighted imaging (DWI). Within 2 min after intravenous injection of gadolinium contrast agent (0.2 mL/kg), the first postcontrast images were acquired, followed by five subsequent postcontrast images were acquired. Axial DWI scans were acquired with two b-values (0 and 1,000 s/mm^2^). All patients had undergone core biopsy or surgery of the abnormal area. The final histopathological results were all recorded.

### 2.3 Region of interest segmentation

**Figure 2:**
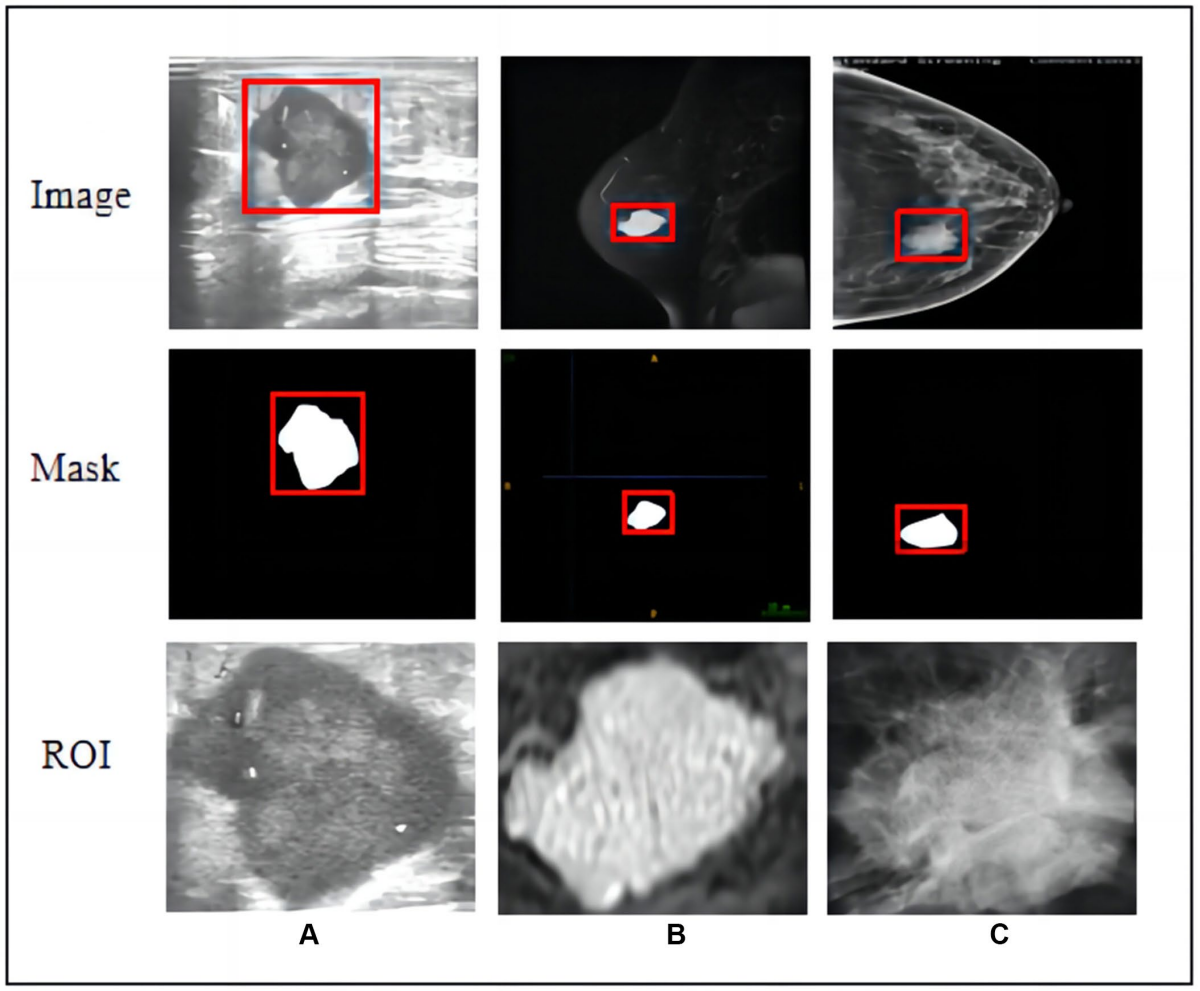
Raw images, hand-crafted masks, and cropped ROIs of three modal images. **(A)** Ultrasound, **(B)** T2-weighted magnetic resonance imaging, and **(C)** mammography (MG) craniocaudal view.

Primary breast tumours were selected for region of interest (ROI) segmentation on the largest layer of the tumour. Two radiologists and one diagnostic ultrasound physician with extensive experience (reader 1 with 12 years, reader 2 with 10 years, and reader 3 with 12 years) in breast imaging diagnosis manually delineated each ROI along the tumour margin from the first to the last layer of the whole tumour using ITK-SNAP software (version 3.80). They completed ROI segmentation under the supervision of a senior radiologist with 30 years of experience in breast imaging diagnosis. The radiologists were blinded to the histopathological information of the malignant breast tumours and benign tumours from the US, MG-CC, and MRI-T2W images. We traced abnormal areas in these images and attempted to delineate the burr at the edge of each tumour as completely as possible. All lesion images were included, as shown in Figure 2.

### 2.4 Feature extraction and selection

A total of 108 radiomic features were extracted using the PyRadiomics (3.0.1) open-source Python package. In this study, the following features were extracted: first-order statistics (FOSs), shape-based 2D (S-2D/3D) features, grey-level co-occurrence matrices (GLCMs), grey-level run length matrices (GLRLMs), grey-level size matrices (GLSZMs), neighbourhood grey tone difference matrices (NGTDMs), and grey-level dependence matrices (GLDMs) (28). Feature selection and fusion techniques were applied to reduce dimensionality and integrate complementary information. The Mann–Whitney U-test and Spearman’s rank correlation coefficient were used to determine the statistical significance and repeatability of the features, respectively. Finally, the least absolute shrinkage and selection operator (LASSO) regression model was used to construct the feature signature for the entire dataset.

**Figure 3:**
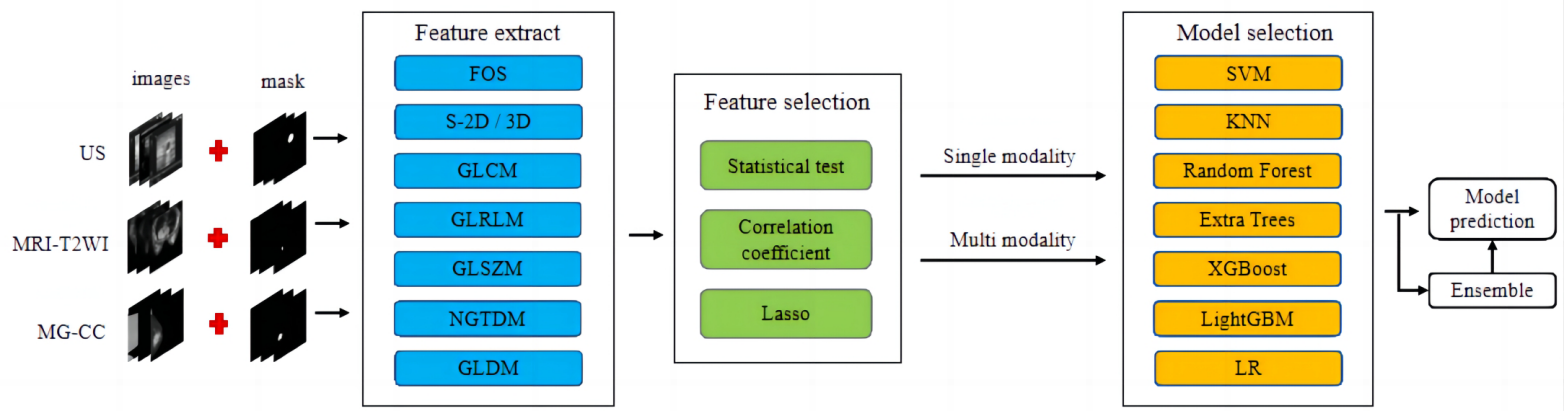
Workflow of conventional radiomics from multimodal data. We extracted conventional radiomic features from US, MR, and MG-CC images. Feature selection and fusion techniques were applied to reduce dimensionality and integrate complementary information. The classification model was constructed using seven machine learning algorithms.

Classification models for single-mode and multimodal fusion were established from multimodal imaging (MG-CC, US, and MRI-T2WI). The classification model was then constructed using different strategies, including support vector machine (SVM), K-nearest neighbour (KNN), random forest (RF), extremely randomised trees (ExtraTree), extreme gradient boosting (XG Boost), light gradient boosting machine (LightGBM), and logistic regression (LR), and the optimal fusion method was selected. The workflow for classification model construction is shown in Figure 3.

**Figure 4:**
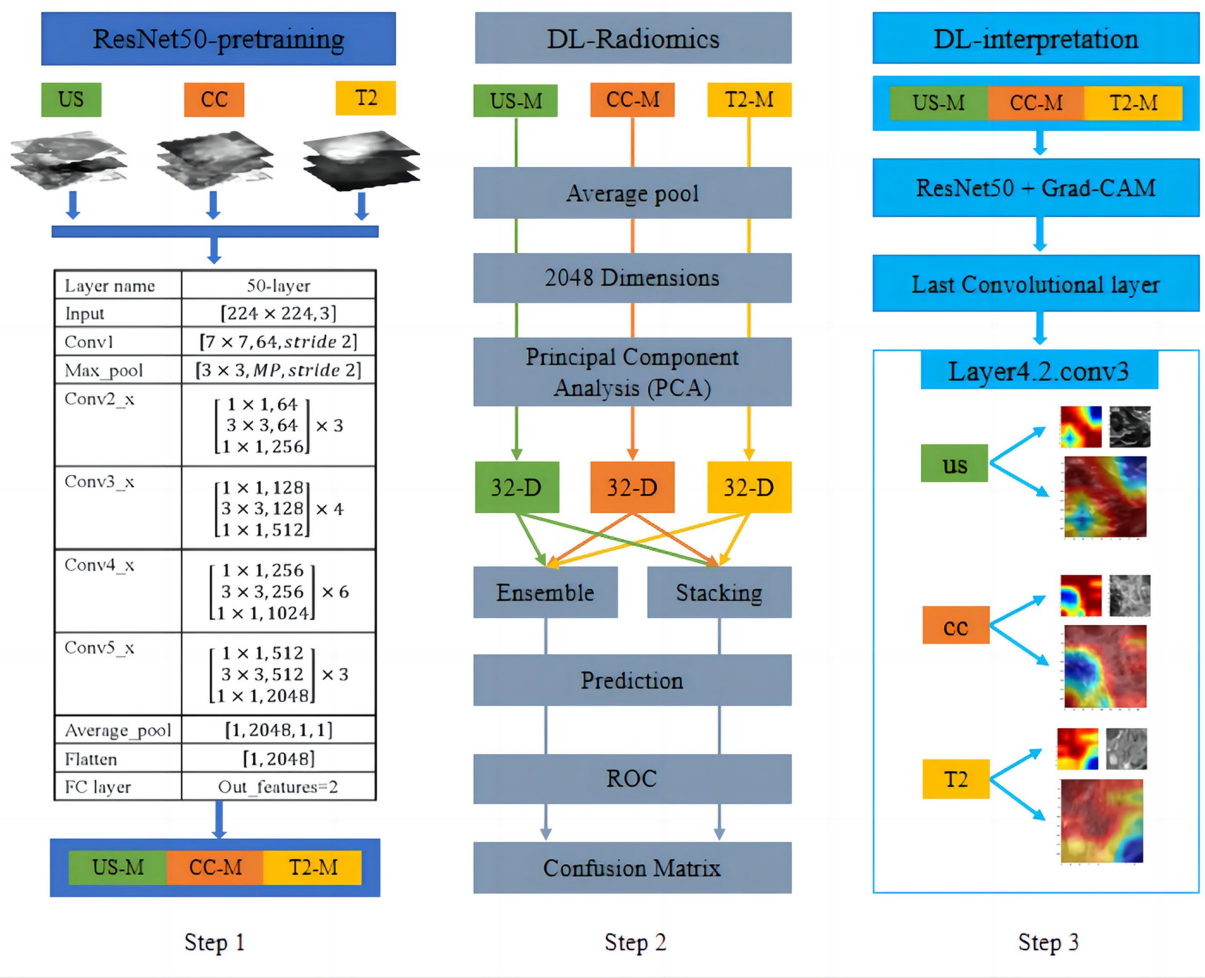
Workflow of transfer learning from multimodal data (Step 1 and Step 2) and visualisation of the CNN decision process (Step 3). For US, MG-CC, and MRI-T2WI images, we employed transfer learning using a pretrained ResNet-50 model. After model training, deep features were extracted from the average pooling layer and reduced in dimensionality with PCA. A classification model was then constructed using two different late-fusion strategies. For model interpretability, Grad-CAM was utilised to visualise and explain the validity of the CNN models.

For US, MG-CC, and MRI-T2WI multimodality imaging, in terms of deep learning, we used a pretrained ResNet-50 model to perform transfer learning tasks on rectangular ROI images acquired from the three imaging modes, as shown in Figure 4 (Step 1). Specifically, the convolution layer parameters of the ResNet-50 model were fixed, and the output of the fully connected layer was 2. During the model training stage, the optimal parameter settings (batch size = 32, learning rate = 0.001, epochs = 200, and optimiser = sgd) were obtained through hyperparameter fine-tuning. Next, we input the images from the three imaging modes into their respective optimal models and derived the deep feature values of the average pooling layer. Since the size of the feature map of the pooling layer was fixed, the number of dimensions of the deep feature values for all the modes was 2048. For feature selection, the PCA algorithm was used to reduce the dimension of the depth feature value. The classification model was then established using different strategies, as shown in Figure 4 (Step 2). For model interpretability, Grad-CAM was utilised to visualise and explain the validity of the multimodal models, as shown in Figure 4 (Step 3).

### 2.5 Feature fusion

**Figure 5:**
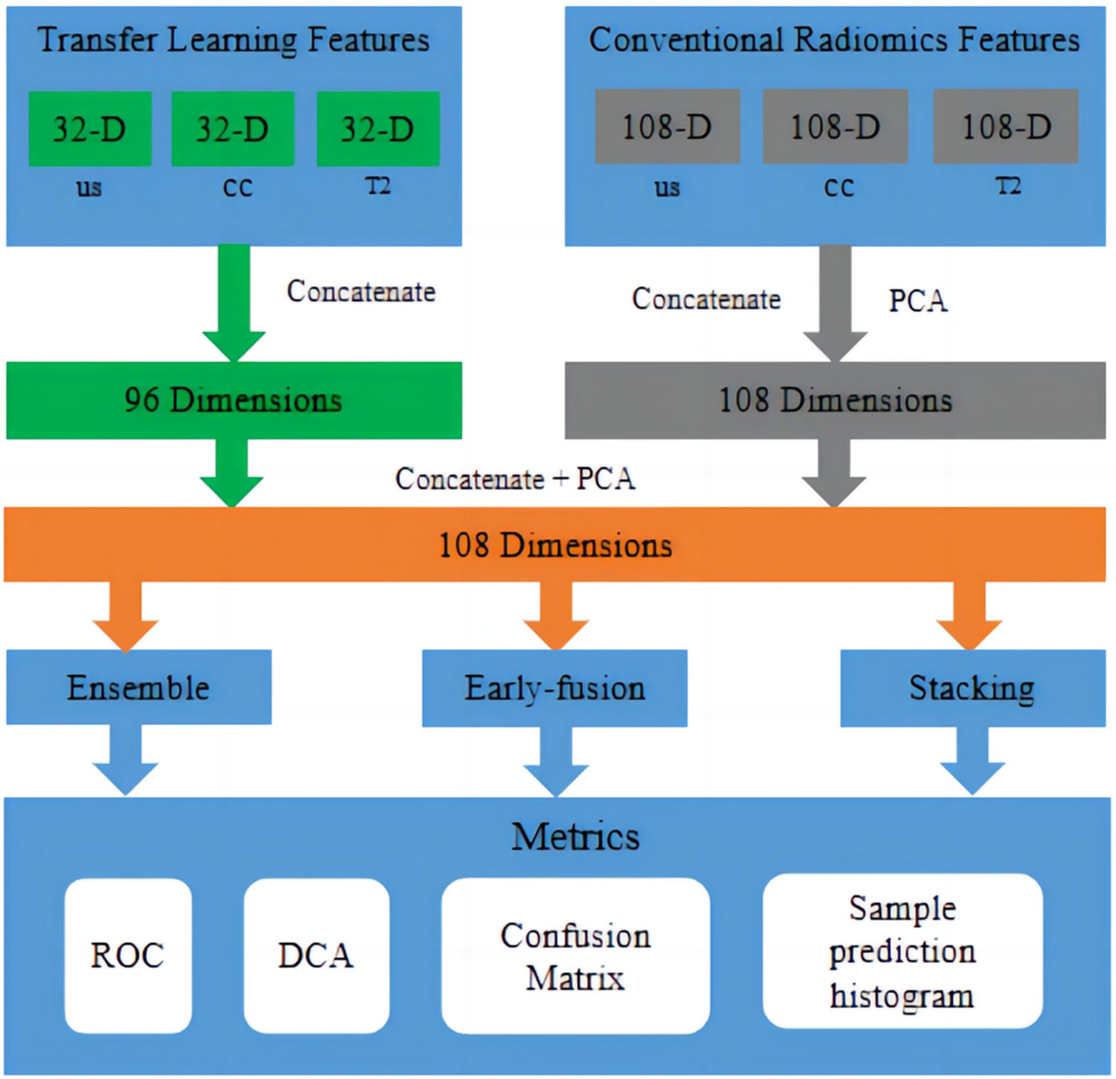
Fusion workflow of deep features and conventional radiomic features from multimodal data. The feature fusion methods were divided into early fusion and late fusion (ensemble and stacking) approaches. In early fusion, features from different modalities were concatenated before modelling to create an integrated feature representation as input to the classifier. In late fusion, separate models were built on each modality first, and then, their outputs were combined via ensemble or stacking techniques to produce final predictions.

The fusion workflow of deep features and conventional radiomic features from multimodal data were below: The feature fusion methods were divided into early fusion and late fusion (ensemble and stacking) approaches. For early fusion, features from different modalities were concatenated before modelling to create an integrated feature representation as input to the classifier. For the ensemble approach, accuracy-weighted average integration based on softmax normalisation weighting was used. For the stacking approach, separate models were first built on each modality, and then their outputs were combined via the ensemble method. Stacking involved using a machine learning model to fuse the results from the training and testing sets and using another machine learning algorithm for classification, as shown in Figure 5.

### 2.6 Evaluation indicators

The model performance evaluation adopted four evaluation metrics, namely accuracy, sensitivity, specificity, and AUC value. Accuracy refers to the proportion of correctly classified samples to the total number of samples. Sensitivity represents the proportion of correctly classified positive samples to the actual number of positive samples. Specificity represents the proportion of correctly classified negative samples to the actual number of negative samples. The AUC is the area under the ROC curve, and the ROC curve is the curve obtained by plotting the True Positive Rate on the Y-axis and the False Positive Rate on the X-axis. The value of AUC ranges from 0.5 to 1, and the higher the AUC, the better the performance of the classifier. TP is the number of true-positive results, FP is the number of false-positive results, TN is the number of true-negative results, and FN is the number of false-negative results.


(1) $$\text{Accuracy}=\frac{\mathrm{TP}+\mathrm{TN}}{\mathrm{TP}+\mathrm{TN}+\mathrm{FP}+\mathrm{FN}}$$

(2) $$\text{Sensitivity}=\frac{\mathrm{TP}\;}{\mathrm{TP}+\mathrm{FN}}$$

(3) $$\text{Specificity}=\frac{\mathrm{FP}}{\mathrm{FP}+\mathrm{TN}}$$

## 3 Results

### 3.1 Clinical characteristics

In this study, 322 female patients with a mean age of 50.48 ± 11.57 years were enrolled. The patients were divided into a training set (257 patients) and a test set (65 patients). A total of 112 benign breast tumours and 210 malignant breast tumours were included. The clinicopathological data and corresponding multimodal imaging data resulted in 6440 data points. No significant difference in clinical features was noted amongst the cohorts (*p* > 0.05), as shown in Table 1.

**Table 1:** Characteristics of breast tumours in this study.

Characteristics	Training (*n* = 257)	Testing (*n* = 65)	Values	*p*
Menstrual status	89 (34.6%)	23 (35.4%)	*χ*2 = 3.078	0.079
Age (years)	50.31 ± 11.57	51.08 ± 10.72	*t* = 0.486	0.627
Diameter (mm)	19.94 ± 11.27	22.78 ± 10.01	*t* = 1.476	0.141
CA-153	19.76 ± 8.97	20.52 ± 10.27	*t* = 0.593	0.554
BI-RADS category			*χ*2 = 6.080	0.108
1–3	57 (22.2%)	24 (36.9%)	–	–
4(4a,4b,4c)	138 (53.7%)	28 (43.1%)	–	–
5	44 (17.1%)	8 (12.3%)	–	–
6	18 (7.0%)	5 (7.7%)		
Pathology			*χ*2 = 0.087	0.768
Benign	89 (34.6%)	23 (35.4%)	–	–
Malignant	168 (65.4%)	42 (64.6%)	–	–

### 3.2 Radiomic model for multimodal imaging

A total of 108 groups of feature values from US, MRI-T2WI, and MG-CC rectangular ROI images were extracted. After feature selection, we retained 42 sets of feature values for the training of the machine learning model. As shown in Figure 3, the experiments were divided into a single-mode radiomic model, a prefusion model (two-mode image fusion model and three-mode image fusion model), and an ensemble fusion modal method. For both single-mode features and multimode fusion features, seven classifiers were tested in the experiment, and finally, the optimal classification model was obtained. For the generation of classification models, 20% of the images were randomly selected for testing, and the other 80% were selected for training. Notably, when training the first classification model, we set up random seeds to fix the instances of the training set and the test set. The established training set and test set ensured the consistency of training and testing of all classification models and thus the fairness of model evaluation.

The combined modalities integrating multimodal imaging (MG-CC, US, and MRI) showed good validity and stability. We described the diagnostic indices of the different modalities for all patients in the primary cohort and validation cohorts. Table 2 and Figure 6A show the evaluation performance of the optimal classification model under different traditional image radiomic feature sets. With respect to the conventional radiomic features, for the single-modal images, MRI-T2WI achieved the best accuracy (80.0%) and an AUC of 0.785 [0.674–0.915]. US had the best sensitivity of 90.4% [81.5–99.3%]. MG-CC had the best specificity of 82.6% [67.1–98.0%] (lines 1 to 3). Amongst the two multimodal methods, US+MRI had the highest AUC of 0.858 [0.763–0.952] and a specificity of 78.2% [61.4–95.1%] (lines 4 to 6). For the three-mode imaging method, the highest accuracy was 84.3%, the AUC was 0.812, the sensitivity was 95.2% [88.7–100.0%], and the specificity was 63.6% (line 7). The ensemble fusion modal method performed the best, with an accuracy of 89.2%, an AUC of 0.942 [0.886–0.996], a sensitivity of 85.7%, and a specificity of 95.6% [87.3–100.0%] (line 8).

**Table 2:** Results of radiomic classification utilising conventional features.

Methods	Accuracy	AUC	Sensitivity	Specificity	Classifier
US	0.784	0.707 [0.555–0.858]	0.904 [0.815–0.993]	0.565 [0.362–0.767]	SVM
MR	0.800	0.795 [0.674–0.915]	0.857 [0.751–0.962]	0.695 [0.507–0.883]	SVM
MG	0.753	0.748 [0.612–0.883]	0.714 [0.577–0.850]	0.826 [0.671–0.980]	XGBoost
US+MR	0.815	0.858 [0.763–0.952]	0.833 [0.720–0.946]	0.782 [0.614–0.951]	SVM
US+MG	0.692	0.718 [0.578–0.857]	0.642 [0.497–0.787]	0.782 [0.614–0.951]	LightGBM
MR + MG	0.815	0.746 [0.603–0.889]	0.881 [0.783–0.978]	0.727 [0.507–0.883]	XGBoost
US+MR + MG	0.843	0.812 [0.693–0.929]	0.952 [0.887–1.000]	0.636 [0.435–0.837]	XGBoost
Ensemble	0.892	0.942 [0.886–0.996]	0.857 [0.751–0.962]	0.956 [0.873–1.000]	SVM + LightGBM#

# denotes the classifiers (SVM + LightGBM), and [] represents the 95% confidence intervals (CI).

**Figure 6:**
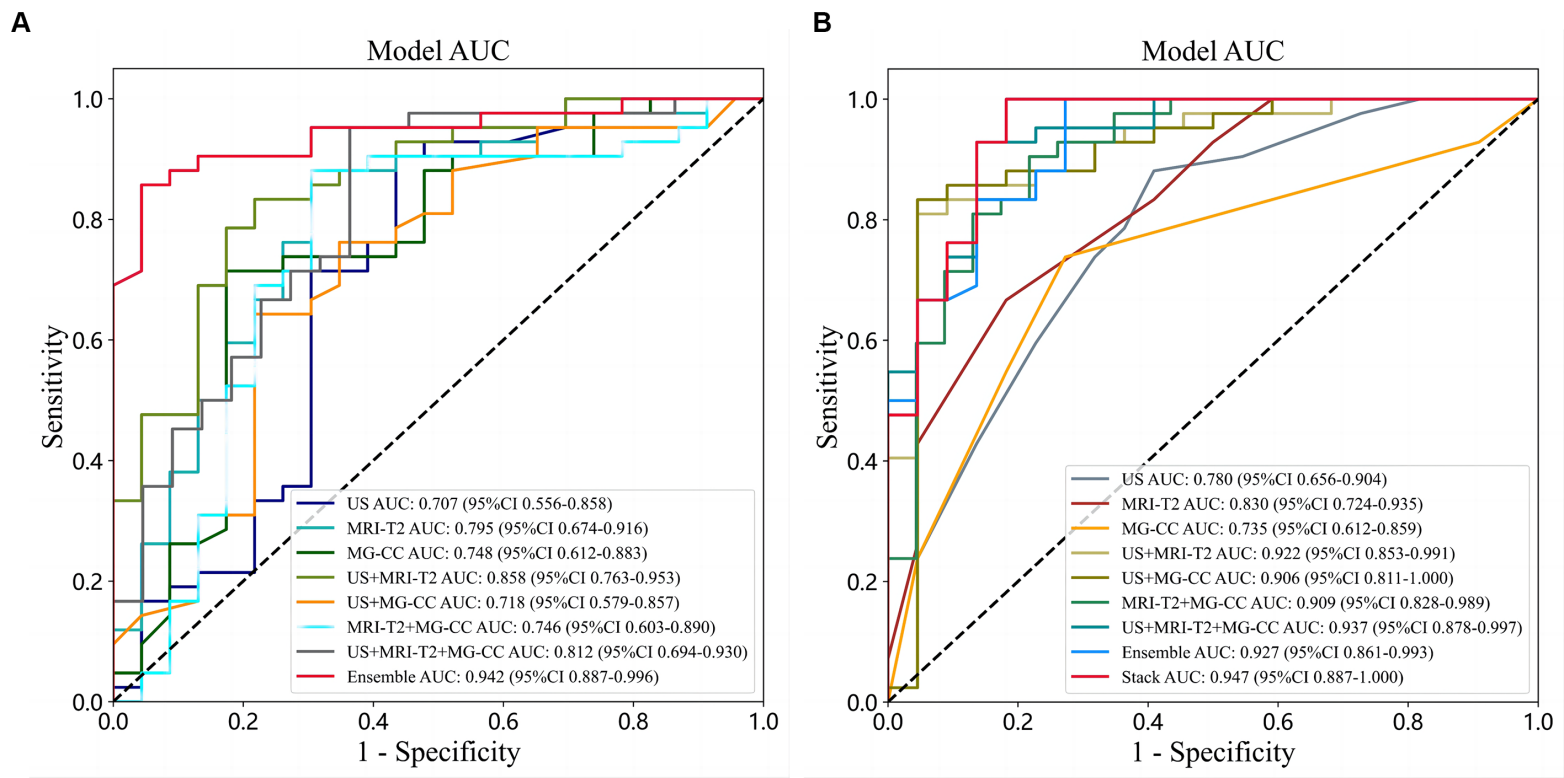
Comparison of ROC curves under different classifiers with different sources of features. **(A)** Conventional radiomic features; **(B)** deep features from transfer learning.

### 3.3 Deep learning models for multimodal imaging

In summary, we used the pretrained ResNet-50 model to extract 2048 sets of feature values from US, MRI-T2WI, and MG-CC rectangular ROI images, as shown in Figure 4 (Step 1). The difference was that feature selection with PCA was used for dimension reduction. The experiment reduced the eigenvalue of each mode to 32 dimensions. In terms of the model, we generated single-mode, multimode, prefusion, and postfusion classification models, as shown in Figure 4 (Step 2). Similarly, seven classification models were tested to determine the optimal classifier. In addition, the experimental setup was also consistent with that described above.

The performance of the deep features from the transfer learning model, when combined with multimodal imaging, outperformed that of the single-mode models, as shown in Table 3 and Figure 6B. For the single-mode images, US achieved the best accuracy and sensitivity of 78.1 and 88.0%, respectively [78.3–97.8%]. MRI had the best AUC and specificity (0.830 [0.723–0.935] and 81.8 [65.7–97.9%], respectively) (lines 1–3). For the two multimodal imaging methods, the accuracies were 85.9, 87.5, and 86.1%; the AUCs were 0.922, 0.906, and 0.909; the sensitivities were 80.9, 83.3, and 90.4%; and the specificities were 95.4, 95.4, and 78.2%, respectively. US+MR and US+MG had the same specificity of 95.4% [86.7–100.0%] (lines 4–6). For the three multimodal imaging methods, the highest accuracy was 90.6%, the AUC was 0.937 [0.877–0.996], the sensitivity was 92.8% [85.0–100.0%], and the specificity was 86.3% (line 7). Overall, the postfusion model’s performance was better than that of the multimodal models. The ensemble model had an accuracy of 90.6%, an AUC of 0.927, a sensitivity of 100.0%, and a specificity of 72.7% (line 8). The stacking model performed best, with an accuracy of 93.7%, an AUC of 0.947 [0.877–1.000], a sensitivity of 100.0% [99.9–100.0%], and a specificity of 81.8% (line 9).

**Table 3:** Results of transfer learning classification utilising deep features.

Methods	Accuracy	AUC	Sensitivity	Specificity	Classifier
US	0.781	0.780 [0.655–0.903]	0.880 [0.783–0.978]	0.619 [0.385–0.796]	RF
MRI	0.719	0.830 [0.723–0.935]	0.667 [0.524–0.809]	0.818 [0.657–0.979]	ExtraTrees
MG	0.734	0.735 [0.611–0.859]	0.738 [0.605–0.871]	0.761 [0.541–0.913]	KNN
US+MR	0.859	0.922 [0.852–0.991]	0.809 [0.690–0.928]	0.954 [0.867–1.000]	XGBoost
US+MG	0.875	0.906 [0.811–1.000]	0.833 [0.720–0.946]	0.954 [0.867–1.000]	SVM
MR + MG	0.861	0.909 [0.828–0.989]	0.904 [0.815–0.993]	0.782 [0.614–0.951]	SVM
US+MR + MG	0.906	0.937 [0.877–0.996]	0.928 [0.850–1.000]	0.863 [0.720–1.000]	XGBoost
Ensemble	0.906	0.927 [0.861–0.992]	1.000 [0.999–1.000]	0.727 [0.541–0.913]	SVM+KNN+LightGBM#
Stacking	0.937	0.947 [0.887–1.000]	1.000 [0.999–1.000]	0.818 [0.657–0.979]	XGBoost

# denotes the classifiers (SVM + KNN + LightGBM).

### 3.4 Deep learning radiomic models for multimodality imaging

The classification model with both conventional image radiomic features and deep learning features showed robust performance. We tried to integrate the conventional image radiomic features and deep learning features from multimodal imaging of breast tumours and further improve the performance of the classification model.

The deep learning feature values of US, MRI-T2WI, and MG-CC were spliced in the same dimension. Figure 5 shows the specific process of feature fusion. In traditional image radiomics, after the three sets of eigenvalues are spliced in the same dimension, the number of dimensions is reduced to 108 according to PCA. After the two types of features were generated, we repeated the above operation, first splicing and then PCA dimension reduction. Finally, we obtained 108-dimensional features containing 51 sets of deep feature values and 57 sets of traditional image radiomic feature values. The 108 sets of features represented a valid feature set for each patient and formed the basis for our classification model. The experiment implemented three fusion methods—early fusion, ensemble, and stacking—for the classification model. Research has shown that the radiomic and deep features of these multimodal images play a decisive role in the final performance of the model (Figure 6).

**Figure 7:**
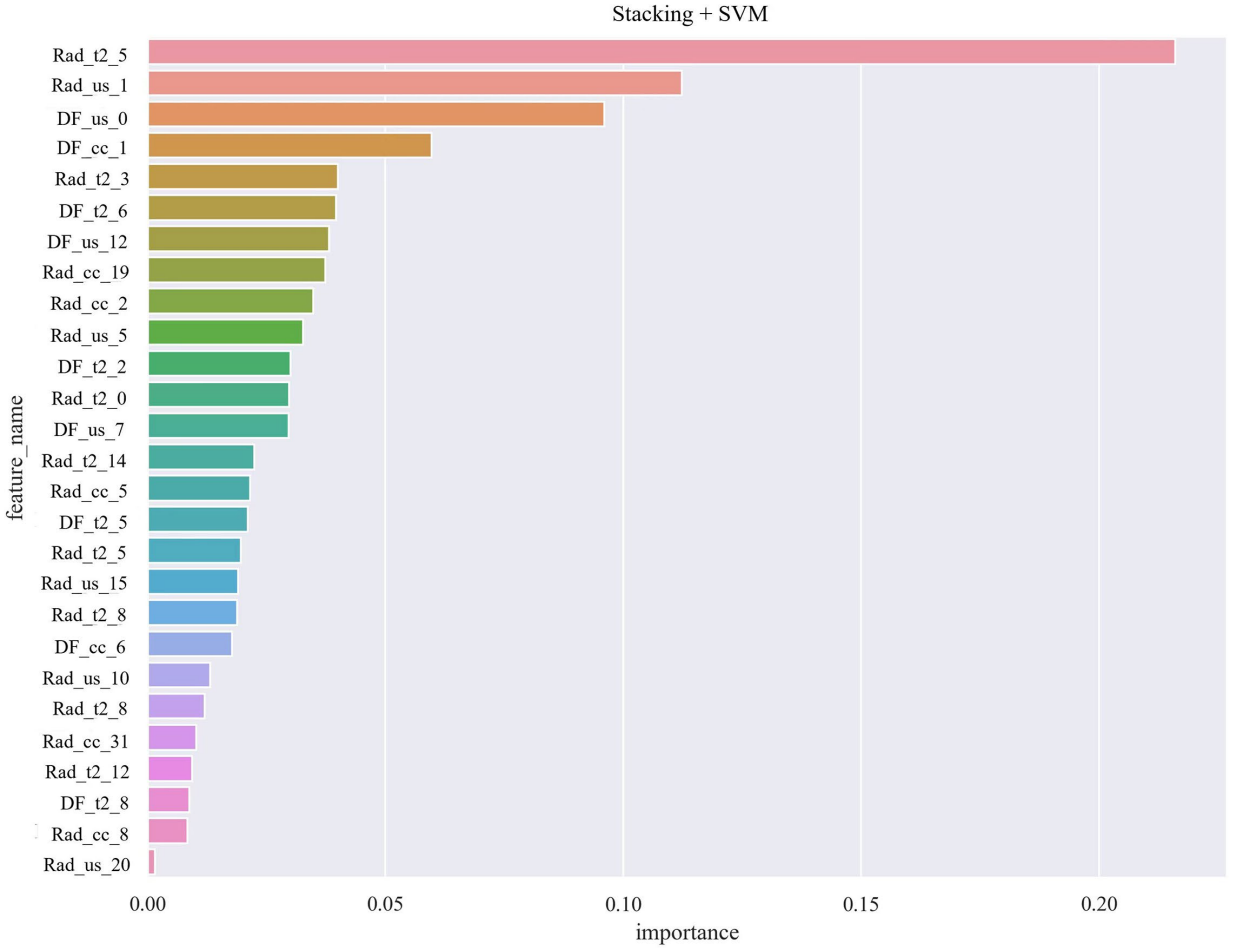
Radiomic and deep features of multimodal images role in the final performance of the model.

Table 4 shows the performance evaluation indices of the three fusion models. We noted that the SVM classifier used in the stacking model achieved the best overall performance, yielding the highest accuracy, AUC, and specificity of 0.968, 0.997 [0.990–1.000], and 1.000 [0.999–1.000], respectively (Figure 7).

**Table 4:** Feature fusion results of conventional radiomic features and deep features from transfer learning.

Methods	Accuracy	AUC	Sensitivity	Specificity	classifier
Rad + DF	0.953	0.986 [0.966–1.000]	1.000 [0.999–1.000]	0.863 [0.720–1.000]	SVM
Ensemble	0.968	0.994 [0.982–1.000]	0.976 [0.930–1.000]	0.954 [0.867–1.000]	SVM+XGBoost+LightGBM#
Stacking	0.968	0.997 [0.990–1.000]	0.952 [0.887–1.000]	1.000 [0.999–1.000]	SVM
VGG-19 (29)	0.846	0.867 [0.775–0.959]	0.938 [0.850–1.000]	0.695 [0.507–0.883]	Softmax
GoogLeNet (30)	0.828	0.807 [0.678–0.935]	0.928 [0.863–0.952]	0.636 [0.435–0.837]	Softmax
ResNet-101 (31)	0.796	0.770 [0.640–0.899]	0.952 [0.827–0.987]	0.500 [0.291–0.708]	Softmax
Inception-v3 (32)	0.875	0.892 [0.803–0.980]	0.952 [0.887–1.000]	0.727 [0.541–0.913]	Softmax

In addition, we compared against existing deep learning classification models. Rad+DF represents fused radiomic and deep features. # represents the classifiers (SVM + XGBoost + LightGBM).

### 3.5 Comparison with different classification models

**Figure 8:**
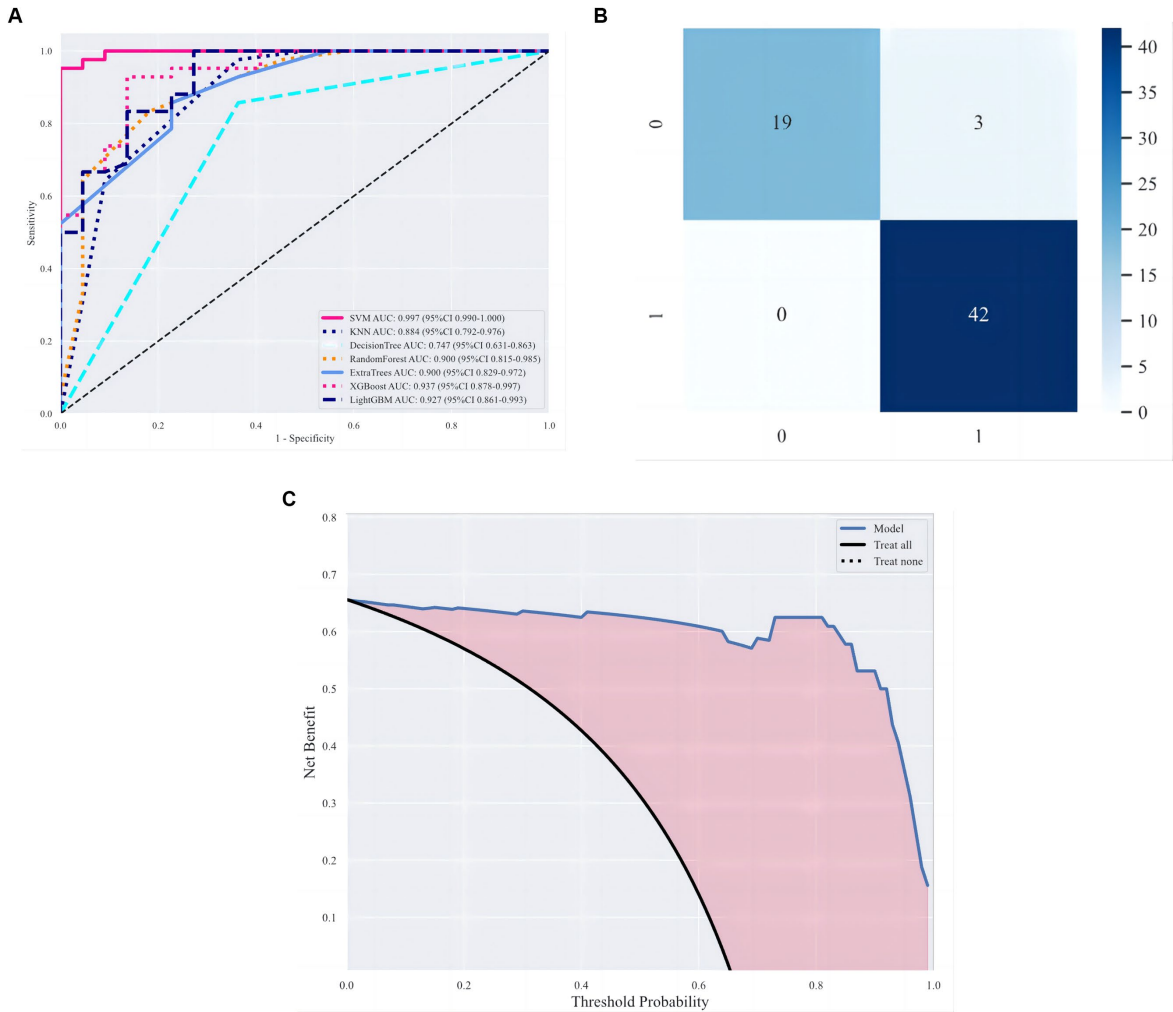
Evaluation of classification models. **(A)** ROC curves under different classifiers. **(B, C)** Optimal classification model evaluation, namely, stacking, including confusion matrix and decision curve analysis (DCA).

By setting random seeds, we fixed the training sets and test sets of cases for the deep learning radiomic model (stacking). This study compared benign and malignant breast tumour classification models based on VGG19 (29), GoogLeNet (30), ResNet-101 (31), and Inception-v3 (32). Specifically, the same training set was used for model migration training and fixed convolution layer and modified fully connected layer parameters (the fully connected layer parameter was set to 2). After the model was generated, the same test set was used for the performance evaluation. Table 4 shows the classification results of the deep learning radiomic models and existing deep learning models. The experiments showed that the deep learning image model, which combined traditional imaging radiomic and deep learning features, was superior to the model based on deep learning in the classification of benign and malignant breast tumours, as shown Figure 8.

## 4 Discussion

Breast cancer shows profound disease heterogeneity, metastasis, and therapeutic resistance and is a leading cause of cancer-related mortality in women. The accuracy and sensitivity of diagnostic tools for differentiating breast tumours need to be further improved, although several diagnostic methods have been developed. Compared to a traditional radiomic model and deep learning feature model, the deep learning radiomic model showed better performance in the classification of benign and malignant breast tumours (33). In our study, we compared a traditional radiomic model, a deep learning model, and a deep learning radiomic model for multimodal imaging. The experimental results showed that the deep learning radiomic fusion model of multimodal imaging exhibited an outstanding performance in distinguishing between benign and malignant breast tumours and achieved the best classification performance. We obtained an AUC of 0.937 with the multimodal model with deep features and transfer learning. With the support of multimodality imaging, the model integrating traditional imaging radiomic and deep learning eigenvalues could more accurately capture key information from the tumour images. Therefore, this model improved the accuracy and robustness of classification.

Hu et al. (34) developed a computer-aided diagnosis method based on dynamic contrast-enhanced (DCE) and T2-weighted MR sequences. The study classified lesions as benign or malignant using support vector machine (SVM) classifiers, and the area under the curve (AUC) of the multiparametric schemes was 0.86 for classifier fusion. The best result was obtained with the feature fusion method. Compared with the prefusion, postfusion added more features into analysed information, so it got the best performance of all the modals.

Huang et al. (35) constructed a deep learning radiopathomic model based on preoperative US images and haematoxylin and eosin (H&E)-stained biopsy slide feature fusion. The deep learning radiopathomic model yielded high performance, with an AUC of 0.929, outperforming the deep learning radiomic model based only on US images and the deep learning pathomic model based only on WSIs. Their study achieved good diagnostic efficacy, which was superior to that of the MG and MRI modalities alone, whilst our study focussed on US, MG, and MRI multimodal and obtained better performance than single-modal or two-fused modals. We also obtained a high AUC of 0.937, similar to that reported by Huang using H&E staining. Therefore, in the future with the mulitmodal image DLR, we may achieve a non-invasive means of examination, aimed at reducing the need for breast mass biopsy.

Based on the performance of the three models, both the deep radiomic model and the feature fusion model outperform the traditional radiomic models in classifying benign and malignant breast tumours. We attribute this to the incorporation of deep feature values into the traditional radiomic features. To assess the efficacy of the deep feature values, the final convolutional layer of the ResNet-50 model was visualised using the Grad-CAM method, as depicted in Figure 4 (Step 3). The visualisation reveals that the deep feature values contributing to the decision-making are distributed within and around the tumour. We observed that the highlighted areas on the heatmap align with those observed by clinicians, underscoring the significance of integrating deep feature values into traditional radiomic features.

The excellent performance of the deep learning radiomic model provides important technical support and guidance for the early diagnosis and treatment of breast tumours. The advantage of deep learning radiomic modalities in breast tumour classification could not only be reflected in the classification performance but also in the full use of multimodal imaging. The fusion of multimodal imaging could provide more comprehensive and multidimensional information for the model such that the model had more diagnostic value and clinical application prospects. Therefore, we believe that the deep learning radiomic imaging model has the best performance in distinguishing benign and malignant breast tumours and plays an important role in the field of medical imaging.

Previous studies have also explored the use of radiomic models and DLR nomograms with promising results. For example, Gao et al. achieved an AUC of 0.82 with a radiomic model using combined craniocaudal + lateral oblique MG features (36). Zhang et al. (37) developed an ultrasound-based DLR nomogram that showed excellent performance in predicting axillary lymph node load. The AUCs of the training and test sets were 0.900 and 0.821, respectively. In our study, in the field of traditional radiomic models, the integrated features in the ensemble model showed better overall performance than the single-mode models. The advantage of this multimodal fusion was not only the integration of information from different imaging modes but also the exquisite design of the ensemble model. The ensemble model seamlessly integrated information from various imaging modes, making full use of the advantages of each mode, thus improving the overall classification performance. Classifiers such as SVM and LightGBM were selected not only because of their applicability in processing multimodal data but also based on their performance and stability in different situations. Through this clever combination, ensemble models were able to maintain high accuracy whilst maintaining modal robustness and generalisability. In the forecasting process, the ensemble model adopted the weighted voting strategy, which synthesised the opinions of various classifiers, effectively reducing the error rate and improving the reliability of the classification results. In summary, the application of the ensemble model to the traditional radiomic model showed its unique advantages in integrating multimodal information and improving classification performance.

Chen et al. (38) used deep learning features from DWI-ADC imaging and DCE-MRI to predict axillary lymph node metastasis with high accuracy (AUC = 0.80 and 0.71) in training and testing cohorts, respectively. In our study, amongst the deep learning models, the model with amalgamated deep learning features demonstrated superior performance compared to the single-mode models. The fusion strategy of the stacking method significantly enhanced the performance and robustness of the model compared to the single-mode deep learning feature model, rendering it more competitive in practical applications. Integrating predicted probabilities into feature sets through the stacking model enhanced new stacking relationships and data labelling, providing a novel idea for further optimisation of deep feature models (39, 40). Unlike traditional radiomics, we also used a stacking-based deep learning feature model, which enhanced the classification performance, particularly the stacking model and XGBoost classifier, amongst the various classifiers. The unique advantage of the stacking model is its ability to effectively fuse deep learning features from each mode and achieve more precise classification using efficient classifiers such as XGBoost. Kwon et al. (41) compared the performance of every meta-learner model with a stacking ensemble approach as a supporting tool for breast cancer classification. The study showed that using specific models as a meta-learner resulted in better performance than that of single classifiers. Mohammed et al. (42) took the output of the submodels (base-learners) as input and then merged the input predictions to determine the final prediction, which was better than that of each of the base-classifiers. In this study, we achieved high accuracy and perfect specificity (100%) with the stacking deep learning model, which may benefit from our multimodal images.

Currently, differentiating malignant breast tumours from benign breast tumours is very important for guiding future clinical treatment and avoiding unnecessary biopsies. Although several diagnostic methods have been developed (34, 43, 44), the accuracy and sensitivity of those tools for differentiating breast tumours need to be further improved. Patterns of breast calcifications visible on mammograms may be useful for differentiating between benign and malignant lesions. A radiomic feature analysis revealed several statistically significant correlations of the tumour and near and far regions in mammograms with intensity-based histogram features, edge frequency features, and Fourier-based power-law beta features (45). Yamamoto et al. studied 353 patients and identified 21 MRI features, finding that they correlated with 71% of the gene expression profiles of breast cancer (46). Cai et al. created a deep learning (DL)-based CNN capable of discriminating amongst benign and malignant microcalcifications of radiological features of the breast (47). Two model datasets are commonly used by several authors in the state of the art (48–50). Our study analyzed multimodal imaging (MG, US and MRI) of breast tumor with deep learning model. And we got good diagnostic efficacy, which was superior to single model image (MG, US or MRI) and two models of fused image (MG+MRI, MG+US or US+MRI). Therefore, the deep learning radiomic method has a certain value in the differential diagnosis of breast tumours, and multimodal image data can complement each other. We also found that multimodality methods had a strong advantage when the maximum diameter was less than 1 cm compared to using one or two model images alone.

In the present study, we also detected a noticeable difference in multimodality imaging between DLR and BI-RADS categories 3 and 4, whilst this difference was not detected for BI-RADS categories 5 and 6. The BI-RADS category sometimes varied amongst the model images. MRI images were much more common than US and MG images in our study, especially for BI-RADS 4 and 5. Multimodality imaging provides the best evaluation of the exact BI-RADS category, so multimodality imaging is recommended for diagnosis or surgical consultation for patients in the BI-RADS category 4 or 5. Witowski et al. studied 13,463 patients with breast carcinoma and developed a CNN model based on T1-weighted MR images to generate a three-dimensional (3D) mask of the breast area, achieving the highest sensitivity for BI-RADS 5 (92.5%) and a low value for BI-RADS 3 (33.3%), indicating that BI-RADS 3 represents an uncertain category not only for radiologists but also for DL approaches. This approach also prevents biopsies from yielding benign results in up to 20% of all patients with BI-RADS category 4 lesions (51). Both of the above results showed that the DLR model could serve as a helpful tool in the reporting system to increase the specificity of cancer screening. We still need to further developped broadly accessible, reliable, and accurate multimodality imagings with DLR tools. In this way breast tumor could be detected earlily and get more measures for prevention.

Our study has several limitations: (1) the classification proposed in this study focussed only on the differentiation of benign or malignant breast tumours; thus, it did not accurately distinguish pathological subtypes, which is a topic for future research. (2) In the present study, we utilised only the CC view in the MG and T2 MR images, whilst other piesces of information, such as lateral oblique images from MG and T1-weighted imaging (T1WI), diffusion-weighted imaging (DWI), apparent diffusion coefficient (ADC) images, and DCE-MRI sequences, were not fully analysed. Exploring these additional data may provide more insights into DLR applications. (3) This was a retrospective analysis with a relatively small sample size. For our future study, we plan to use a multicentre external validation dataset and prospective validation to further confirm these findings. (4) Manual segmentation of ROIs on each image slice increases the workload. Further studies should focus on developing deep learning-based segmentation methods for automatic lesion segmentation via multimodal imaging.

## 5 Conclusion

In this study, we demonstrated the potential of integrating deep learning and radiomic features with multimodal images. As a single modality, MRI based on radiomic features achieved greater accuracy than US or MG. The US and MG models achieved higher accuracy with transfer learning than the single-mode or radiomic models. Our findings may contribute to the growing body of research on the use of DLR in breast cancer diagnosis and classification with MG, US, and MRI. The traditional radiomic and depth features of US+MG + MR achieved the highest sensitivity under the early fusion strategy, exhibited higher diagnostic performance, and provided more valuable information for differentiation between benign and malignant breast tumours. By incorporating multimodal images and DLR analysis, we demonstrated the potential for improved accuracy and clinical relevance in distinguishing breast mass characteristics. In future investigations and validation, we plan to employ the designed fusion approach to other medical images, for example, PET/CT or PET/MRI.

## Data availability statement

The raw data supporting the conclusions of this article will be made available by the authors, without undue reservation.

## Ethics statement

The studies involving humans were approved by General Hospital of Northern Theater Command, No. Y(2404)-030. The studies were conducted in accordance with the local legislation and institutional requirements. Written informed consent for participation was not required from the participants or the participants' legal guardians/next of kin in accordance with the national legislation and institutional requirements.

## Author contributions

GL: Conceptualization, Formal analysis, Writing – original draft, Writing – review & editing. RT: Methodology, Writing – original draft. WY: Methodology, Resources, Writing – review & editing. RL: Data curation, Writing – review & editing. DL: Writing – review & editing, Data curation. ZX: Data curation, Writing – original draft. GZ: Conceptualization, Funding acquisition, Project administration, Supervision, Validation, Writing – review & editing.
