# Correction: Deep learning radiomics based on multimodal imaging for distinguishing benign and malignant breast tumours

**DOI:** 10.3389/fmed.2025.1635819

**Published:** 2025-06-23

**Authors:** Guoxiu Lu, Ronghui Tian, Wei Yang, Ruibo Liu, Dongmei Liu, Zijie Xiang, Guoxu Zhang

**Affiliations:** ^1^College of Medicine and Biological Information Engineering, Northeastern University, Shenyang, Liaoning, China; ^2^Department of Nuclear Medicine, General Hospital of Northern Theater Command, Shenyang, Liaoning, China; ^3^Department of Radiology, Cancer Hospital of China Medical University, Liaoning Cancer Hospital and Institute, Shenyang, Liaoning, China; ^4^Department of Ultrasound, Beijing Shijitan Hospital, Capital Medical University, Beijing, China; ^5^Biomedical Engineering, Shenyang University of Technology, Shenyang, Liaoning, China

**Keywords:** deep learning, radiomics, multimodality imaging, breast tumours, deep learning radiomics, MRI, Mammography, Ultrosonography

In the published article, there was an error in [Figure 1. Flowchart of patient recruitment] as published. [A total of 322 patients enrolled in the study(including 122 benign tomors and 210 malignany tumors), Train set (*n* = 257) Benign = 96, malignant = 161].

The corrected [Figure 1. Flowchart of patient recruitment] and its caption [Figure 1. Flowchart of patient recruitment. A total of 322 patients enrolled in the study (including 112 benign tumors and 210 malignancy tumors), with the training set (*n* = 257) consisting of 89 benign tumors and 168 malignant tumors.]

**Figure 1:**
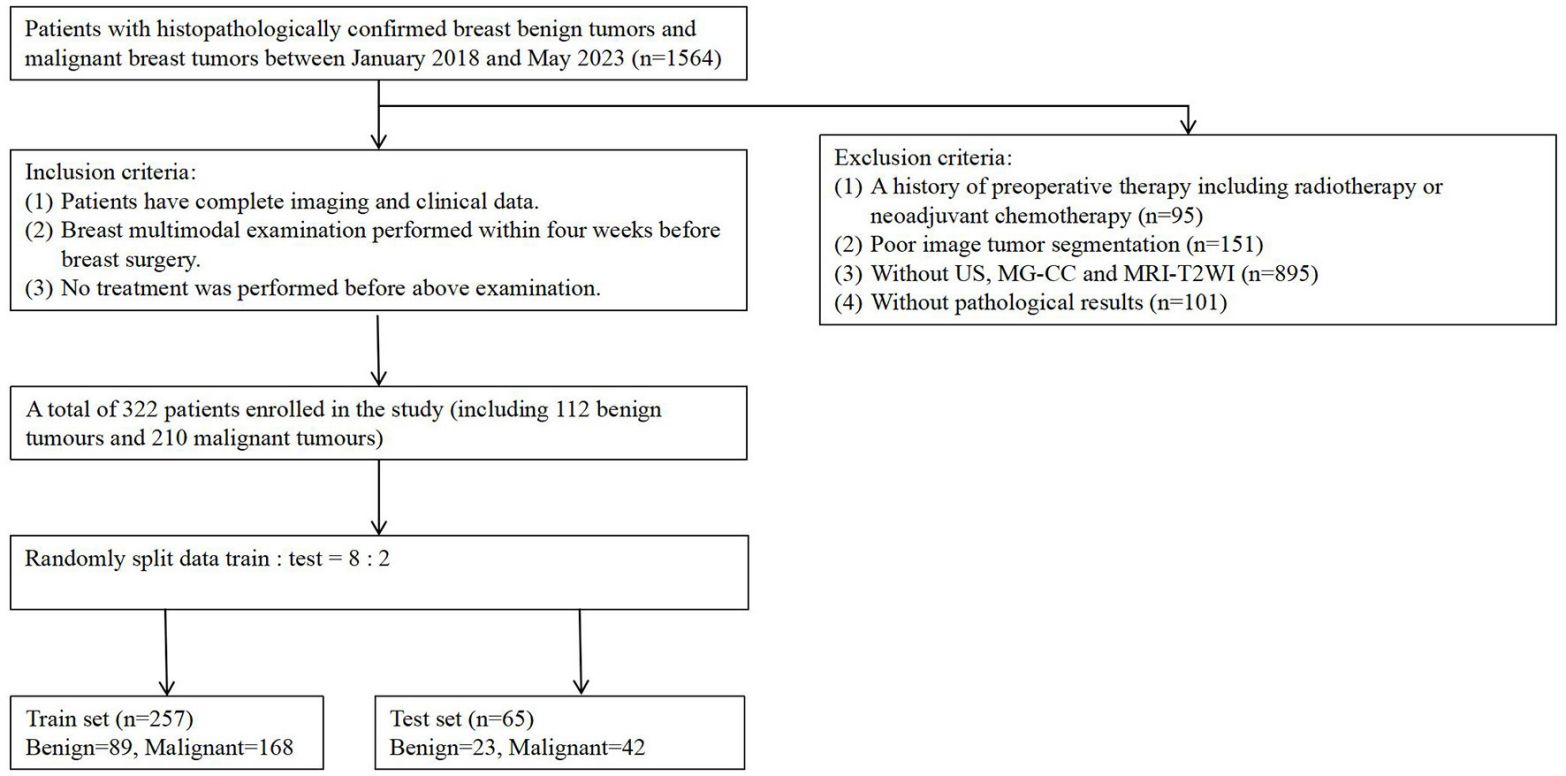
Flowchart of patient recruitment. A total of 322 patients enrolled in the study (including 112 benign tumors and 210 malignancy tumors), with the training set (*n* = 257) consisting of 89 benign tumors and 168 malignant tumors.

In the published article, there was an error in [Table 1, Characteristics of breast tumors in this study: in the training columns, Benign: 96(37.4%), Malignant: 161(62.6%)] as published.

The corrected [Table 1. Characteristics of breast tumors in this study. Benign: 89 (34.6%), malignant: 168 (65.4%)], and its caption [in the training columns] appears below.

**Table 1:** Characteristics of breast tumours in this study.

**Characteristics**	**Training (*n* = 257)**	**Testing (*n* = 65)**	**Values**	***P***
Menstrual status	89 (34.6%)	23 (35.4%)	χ^2^ = 3.078	0.079
Age (years)	50.31 ± 11.57	51.08 ± 10.72	t = 0.486	0.627
Diameter (mm)	19.94 ± 11.27	22.78 ± 10.01	t = 1.476	0.141
CA-153	19.76 ± 8.97	20.52 ± 10.27	t = 0.593	0.554
BI-RADS category			χ^2^ = 6.080	0.108
1–3	57 (22.2%)	24 (36.9%)	-	-
4 (4a,4b,4c)	138 (53.7%)	28 (43.1%)	-	-
5	44 (17.1%)	8 (12.3%)	-	-
6	18 (7.0%)	5 (7.7%)		
Pathology			χ^2^ = 0.087	0.768
Benign	89 (34.6%)	23 (35.4%)	-	-
Malignant	168 (65.4%)	42 (64.6%)	-	-

In the published article, there was an error. In Section *2.1 Patient population* was published with 257 patients (96 with benign breast tumours and 161 with malignant breast tumours) enrolled in the training cohort. The corrected sentence appears below: **[**The training cohort included 257 patients (89 with benign and 168 with malignant breast tumors)]”.

The authors apologize for this error and state that this does not change the scientific conclusions of the article in any way. The original article has been updated.

